# Efficacy and safety of vildagliptin and voglibose in Japanese patients with type 2 diabetes: a 12-week, randomized, double-blind, active-controlled study

**DOI:** 10.1111/j.1463-1326.2010.01222.x

**Published:** 2010-08

**Authors:** Y Iwamoto, A Kashiwagi, N Yamada, S Terao, N Mimori, M Suzuki, H Tachibana

**Affiliations:** 1 Diabetes Center, Tokyo Women's Medical University, School of MedicineTokyo, Japan; 2 Department of Medicine, Shiga University of Medical ScienceShiga, Japan; 3 Department of Internal Medicine (Endocrinology and Metabolism), Graduate School of Comprehensive Human Sciences, University of TsukubaIbaraki, Japan; 4 Novartis Pharma K.K.Tokyo, Japan

**Keywords:** DPP-4 inhibitor, HbA1c, type 2 diabetes mellitus, vildagliptin, voglibose

## Abstract

**Aim:** To confirm the efficacy of vildagliptin in patients with type 2 diabetes (T2D) by testing the hypothesis that glycosylated haemoglobin (HbA1c) reduction with vildagliptin is superior to that with voglibose after 12 weeks of treatment.

**Methods:** In this 12-week, randomized, double-blind, active-controlled, parallel-group study, the efficacy and safety of vildagliptin (50 mg bid, n = 188) was compared with that of voglibose (0.2 mg tid, n = 192) in patients with T2D who were inadequately controlled with diet and exercise.

**Results:** The characteristics of two groups were well matched at baseline. The mean age, body mass index (BMI) and HbA1c were 59.1 years, 24.9 kg/m^2^ and 7.6%, respectively. At baseline, fasting plasma glucose (FPG) and 2-h postprandial glucose (PPG) were 9.01 mmol/l (162.2 mg/dl) and 13.57 mmol/l (244.3 mg/dl), respectively. The adjusted mean change in HbA1c from baseline to endpoint was −0.95 ± 0.04% in the vildagliptin-treated patients and −0.38 ± 0.04% in those receiving voglibose (between-group change = 0.57 ± 0.06%, 95% confidence interval (CI) (−0.68 to −0.46%), p < 0.001), showing that vildagliptin was superior to voglibose. Endpoint HbA1c ≤ 6.5% was achieved in 51% vildagliptin-treated patients compared with 24% patients who were on voglibose (p < 0.001). Vildagliptin also exhibited significantly (p < 0.001) greater reduction compared with voglibose in both FPG [1.34 vs. 0.43 mmol/l (24.1 vs. 7.8 mg/dl)] and 2-h PPG [2.86 vs. 1.1 mmol/l (51.5 vs. 19.8 mg/dl)]. Overall adverse events (AEs) were lower in the vildagliptin-treated patients compared with that in the voglibose-treated patients (61.2 vs. 71.4%), with no incidence of hypoglycaemia and serious adverse events with vildagliptin. Gastrointestinal AEs were significantly lower with vildagliptin compared with that of the voglibose (18.6 vs. 32.8%; p = 0.002).

**Conclusions:** Vildagliptin (50 mg bid) showed superior efficacy and better tolerability compared with voglibose in Japanese patients with T2D.

## Introduction

The prevalence of type 2 diabetes (T2D) is increasing worldwide, and an estimated ∼8.9 million diabetic cases are being reported in Japan [1,2]. Despite major advancement in the treatment for T2D, optimal glycaemic control remains elusive [3]. The undesirable effects associated with many of the available treatment options complicate the treatment of T2D [4,5]. Therefore, novel therapeutic strategies capable of delivering better antihyperglycaemic efficacy with a low frequency of undesirable effects are required.

Voglibose, an *α*-glucosidase inhibitor, is one of the most commonly used oral antidiabetic drug (OAD) in Japan. *α*-Glucosidase inhibitor is prescribed not only as a first-line treatment but also as an add-on to other OADs and insulin [6]. The postprandial glucose (PPG) control exercised by *α*-glucosidase inhibitor is of utmost importance, because this is known to reduce cardiovascular events [7,8]. Voglibose decreases the level of PPG with very low risk of hypoglycaemia, but is associated with frequent gastrointestinal (GI) side effects [9].

The development of dipeptidyl peptidase-IV (DPP-4) inhibitors, such as vildagliptin, has led to the improvement in glycaemic control in T2D by preventing the degradation of the incretin hormones glucagon-like peptide-1 (GLP-1) and glucose-dependent insulinotropic polypeptide. In addition, vildagliptin treatment improves pancreatic *β*-cell and *α*-cell function [10,11], leading to reduced PPG levels. Several clinical trials have showed the efficacy of vildagliptin in patients with T2D either as monotherapy or in combination with metformin [12], thiazolidinedione [13], sulfonylurea [14] or insulin [15] without clinically significant weight gain and with minimal hypoglycaemia. In a 12-week study in Japanese patients with T2D, treatment with vildagliptin was shown to exert dose-dependent glycaemic control and was well tolerated [16]. As treatment with vildagliptin has showed benefits beyond glycaemic control such as lowering of pancreatic *α*-cell glucagon levels [10,17] and preservation of pancreatic *β*-cell function [18,19], vildagliptin may be a better therapeutic option compared with voglibose.

This study aimed to confirm the efficacy of vildagliptin in patients with T2D by testing the hypothesis that the glycosylated haemoglobin (HbA1c) reduction with vildagliptin is superior to that with voglibose after 12 weeks of treatment.

## Methods

### Study Design

This randomized, double-blind, active-controlled, parallel-group study was conducted at 51 centres in Japan. Subsequent to a 2-week run-in period, all eligible patients were randomized (1 : 1) to receive either vildagliptin 50 mg bid or voglibose 0.2 mg tid for 12 weeks.

### Study Population

T2D patients [aged ≥20 years, body mass index (BMI) 20–35 kg/m^2^] with HbA1c of 6.5–10%, fasting plasma glucose (FPG) of <15 mmol/l (270 mg/dl) and those who had not taken OAD for at least 8 weeks before the study entry were enrolled. Patients who received diet/exercise therapy for at least 8 weeks and agreed to maintain it during the course of the study were also eligible. Female patients with non-childbearing potential or those using a medically approved birth control method were included.

Any history of type 1 or secondary forms of diabetes, acute metabolic diabetic complications 6 months before the study, congestive heart failure requiring pharmacological treatment, myocardial infarction, unstable angina, or coronary artery bypass surgery within 1 year, led to exclusion from the study. Patients with renal disease or dysfunction (i.e. elevated serum creatinine levels) and liver disease such as cirrhosis or chronic active hepatitis were also excluded from the study. Concomitant medications with any OADs, chronic corticosteroids (oral or parenteral, >7 consecutive days of treatment) and classes Ia, Ib, Ic or III antiarrhythmic medications were not permitted.

### Study Assessments

The variables HbA1c (measured by HPLC), FPG, fasting insulin, C-peptide and body weight were all measured at screening and at weeks 0, 2, 4, 8 and 12; whereas fasting lipid profile was measured at screening, weeks 0 and 12. Homeostasis model assessment (HOMA)-*β* and HOMA-R were calculated as a function of FPG and insulin, respectively. The standard meal challenge assessment was performed at weeks 0 and 12. For the meal challenge, patients were fasted overnight, and the study drug was administered 5 min before starting a standard 400 kcal breakfast meal (protein, 14.4 g; carbohydrate, 60.5 g; lipids, 9.4 g). Blood samples for the determination of glucose and insulin were obtained at 20 and 0 min before and at 60 and 120 min after the start of the meal.

All the adverse events (AEs) were recorded and assessed for severity and possible relationship to study medication. Patients were provided with and instructed on the use of a self-monitored blood glucose (SMBG) device. Hypoglycaemia was defined as symptoms suggestive of low blood glucose and further confirmed using SMBG measurement <3.1 mmol/l (56 mg/dl) plasma glucose equivalent. Severe hypoglycaemia was defined as any episode; wherein, the patient was unable to initiate self-treatment and required the assistance of another person or hospitalization.

Physical examinations, vital signs, 12-lead ECG and laboratory assessments comprising routine haematology, routine biochemistry and urinalysis were monitored. All laboratory assessments were made by the central laboratory (Mitsubishi Kagaku Bio-Clinical Laboratories, Tokyo, Japan).

### Data Analysis

A sample size of 370 randomized patients (185 per group) was targeted assuming a 10% drop-out rate and a standard deviation of 1. This would provide a 95% power to detect a statistically superior difference of 0.4% from baseline in reducing HbA1c between the two treatment groups using two-sided significance level of 0.05.

The efficacy analyses were performed with data from the full analysis set (FAS) population defined as all the randomized patients who received at least one dose of study drug and had at least one postbaseline assessment. The last observation carried forward (LOCF) method was used for patients who discontinued early. The primary efficacy variable, change in HbA1c from baseline at the study endpoint, was analysed for superiority of vildagliptin 50 mg bid over voglibose 0.2 mg tid. Changes in FPG, fasting plasma lipids and body weight were the secondary efficacy parameters.

Several criteria were prespecified to classify patients as responders to treatment: percentage of patients (i) achieving HbA1c ≤ 6.5%, (ii) experiencing a ≥1.0% reduction in HbA1c, (iii) experiencing a ≥0.7% reduction in HbA1c, (iv) experiencing a ≥0.5% reduction in HbA1c and (v) meeting at least one of the aforementioned criteria in both treatment groups.

The changes from baseline in efficacy variables were analysed using an ancova model, with treatment and pooled centre as the classification factor and baseline as the covariate. The between-treatment differences and the related two-sided 95% confidence interval (CI) for the adjusted mean change for the primary and the secondary efficacy variables were also analysed. Prespecified subanalyses of HbA1c were conducted based on initial (baseline) HbA1c.

Safety assessment was performed on safety (SAF) population and consisted of all subjects who received at least one dose of the study drug and had at least one postbaseline safety assessment. Safety assessments for the incidence of GI AEs were summarized by preferred term for each treatment group, and the overall incidence of GI AEs between both treatment groups was compared using a Fisher's exact test.

### Ethics and Good Clinical Practice

All participants provided written informed consent. The protocol was approved by the independent ethics committee/ institutional review board at each study site, and the study was conducted using Good Clinical Practice in accordance with the Declaration of Helsinki.

## Results

### Patient Disposition and Baseline Characteristics

**Figure 1:**
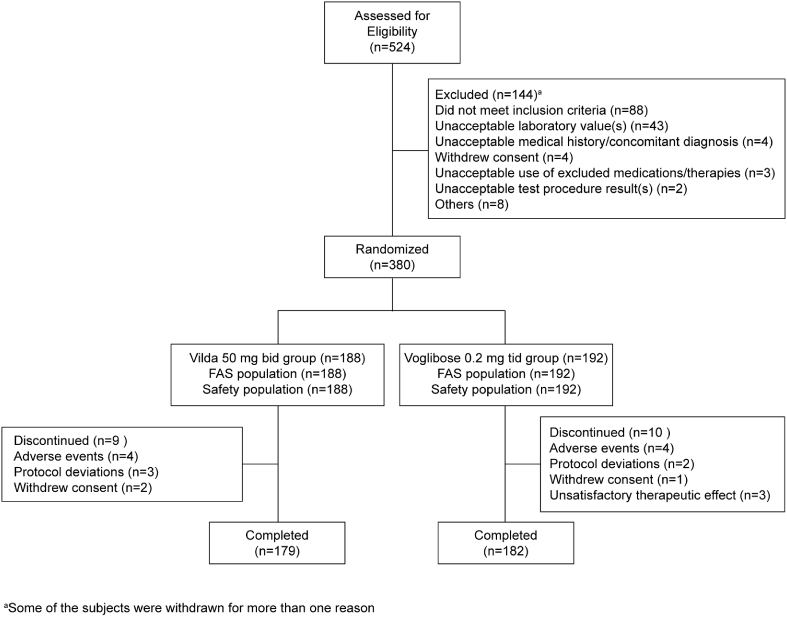
Patients' disposition.

All randomized patients took the study drug (vildagliptin, n = 188; voglibose, n = 192). A comparable proportion of patients completed the study in both treatment groups [vildagliptin: 95.2% (n = 179), voglibose: 94.8% (n = 182)]. Figure 1 summarizes the patient disposition. The number of discontinuations and reasons for discontinuation in both treatment arms were similar. Patient compliance with the study medication was 97.9 and 99.0% in the vildagliptin and voglibose groups, respectively.

The demographic and baseline characteristics of the randomized patients are summarized in Table 1. The groups were well balanced for all the baseline characteristics: mean BMI, 24.9 ± 3.2 kg/m^2^; HbA1c, 7.6 ± 0.9%; FPG, 9.01 ± 1.77 mmol/l (162.2 ± 31.9 mg/dl); 2-h PPG, 13.57 ± 2.98 mmol/l (244.3 ± 53.6 mg/dl). The mean values of age and duration of diabetes were 59.1 ± 10 and 5.4 ± 5.1 years, respectively.

**Table 1:** Baseline characteristics of the randomized population.

Demographic variable	Vildagliptin 50 mg bid N = 188	Voglibose 0.2 mg tid N = 192
Age (years)*	60.3 (±10.48)	58.0 (±9.32)
Age category ≥65 years†	68 (36.2%)	52 (27.1%)
Gender†		
Male	121 (64.4%)	130 (67.7%)
Female	67 (35.6%)	62 (32.3%)
Body weight*	64.4 (±10.8)	66.5 (±10.3)
BMI (kg/m^2^)*	24.8 (±3.05)	25.0 (±3.35)
HbA1c (%)*	7.5 (±0.9)	7.6 (±0.9)
FPG (mmol/l)*	8.9 (±1.7)[160.4(±30.1)]$	9.1 (±1.9)[164(±33.5)]$
1-h PPG (mmol/l)*	15.2 (±2.2)[272.8(±40.1)]$	15.0(±2.4)[270(±43.3)]$
2-h PPG (mmol/l)*,‡	13.6 (±3.0)[244.9(±53.2)]$	13.5(±3.0)[243.8(±54.2)]$
Duration of T2D (years)*	5.1 (±5.1)	5.6 (±5.0)

BMI, body mass index; FPG, fasting plasma glucose; HbA1c, haemoglobin A1c; PPG, postprandial glucose; T2D, type 2 diabetes.

* Mean (±s.d.).

† n (%).

‡ N = 187 for vildagliptin 50 mg bid group.

$ mg/dl.

### Efficacy Results

**Figure 2:**
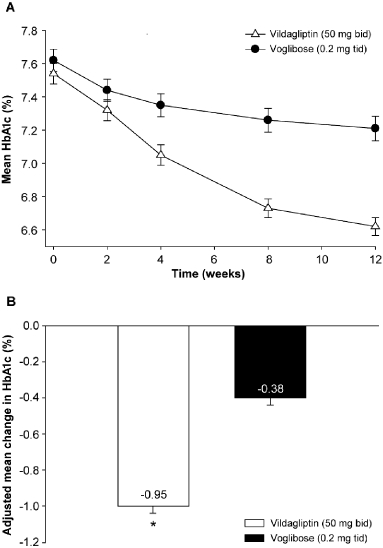
Panel (A): changes in mean (±s.e.) HbA1c during the 12-week treatment with vildagliptin 50 mg bid (open triangles) or voglibose 0.2 mg tid (closed circles). Panel (B): least square mean (±s.e.) change in HbA1c at endpoint under treatment with vildagliptin 50 mg bid (white bar) or voglibose 0.2 mg tid (black bar). *p < 0.001 vs voglibose. HbA1c, haemoglobin A1c.

Figure 2 shows reduction in the HbA1c levels. Greater reduction as early as week 2 was evident with the vildagliptin 50 mg bid group compared with the voglibose 0.2 mg tid group (Figure 2A). At the week 12 endpoint, the adjusted mean of HbA1c reduction from baseline was 0.95 and 0.38% in the vildagliptin and voglibose treatment groups, respectively. The between-treatment difference (vildagliptin–voglibose) in adjusted mean change in HbA1c reduction was significant [0.57 ± 0.06%, 95% CI (−0.68 to −0.46%), p < 0.001], demonstrating that vildagliptin 50 mg bid was superior to voglibose 0.2 mg tid (Figure 2B).

The reduction in HbA1c from baseline was the highest in the >8% category among the subgroups stratified according to baseline HbA1c (vildagliptin: 1.35%; voglibose: 0.49%). The changes in HbA1c were similar in all the other subgroups (baseline BMI, age, gender and glomerular filtration rate) (Table 2).

**Table 2:** Change in HbA1c from baseline at endpoint in subgroups (FAS).

	Vildagliptin 50 mg bid (N = 188)			Voglibose 0.2 mg tid (N = 192)		
Subgroup category	n*	Baseline mean	Change from baseline mean (s.e.)	n*	Baseline mean	Change from baseline mean (s.e.)
Baseline HbA1c (%)						
≤7	65	6.73	−0.56 (0.043)	65	6.71	−0.27 (0.060)
>7, ≤8	71	7.42	−0.95 (0.049)	72	7.49	−0.37 (0.064)
>8	52	8.73	−1.35 (0.094)	55	8.85	−0.49 (0.112)
BMI at baseline						
<25 kg/m^2^	107	7.49	−0.96 (0.056)	106	7.59	−0.37 (0.049)
≥25kg/m^2^	81	7.62	−0.88 (0.063)	86	7.65	−0.38 (0.081)
Age (years)						
<65	120	7.67	−0.93 (0.054)	140	7.67	−0.36 (0.054)
≥65	68	7.31	−0.91 (0.066)	52	7.46	−0.42 (0.080)
Gender						
Male	121	7.55	−0.98 (0.051)	130	7.67	−0.41 (0.060)
Female	67	7.53	−0.81 (0.072)	62	7.51	−0.30 (0.062)
FPG at baseline (mmol/l)						
<8.9 (160 mg/dl)	103	7.10	−0.82 (0.052)	98	7.11	−0.44 (0.046)
≥8.9	85	8.08	−1.05 (0.066)	94	8.14	−0.31 (0.078)
GFR MDRD at baseline [(ml/min)/(1.73 m^2^)]						
Normal >80	170	7.57	−0.92 (0.045)	179	7.61	−0.36 (0.047)
Mild >50 to ≤80	18	7.27	−0.92 (0.090)	13	7.67	−0.48 (0.174)
2-h PPG level at baseline (mmol/l)						
<12.2 (220 mg/dl)	64	6.94	−0.75 (0.055)	61	7.04	−0.40 (0.060)
≥12.2	123	7.86	−1.02 (0.055)	131	7.88	−0.36 (0.060)
No data	1	6.60	0.00			

BMI, body mass index; FPG, fasting plasma glucose; GFR, glomerular filtration rate; MDRD, modification of diet in renal disease; PPG, postprandial glucose.

* n is the number of subjects with observations at both baseline and endpoint.

### Responder Rates

The percentage of responders (target HbA1c ≤ 6.5% at endpoint) was significantly higher with vildagliptin 50 mg bid compared with that to voglibose 0.2 mg tid (50.8 vs. 24.2%, p < 0.001). A higher proportion of patients in the vildagliptin group met the responder criteria of ≥1% reduction in HbA1c (43.1 vs. 15.1%), ≥0.7% reduction in HbA1c (66.5 vs. 31.3%), and a ≥0.5% reduction in HbA1c (83 vs. 44.8%) compared with patients in the voglibose groups. The treatment response in terms of reduction of HbA1c meeting in at least one of the aforementioned criteria was 84% in the vildagliptin group and 51.6% in the voglibose group. In all other definitions, the percentage of responders was significantly higher in the vildagliptin group compared with that of the voglibose group (p < 0.001 in all criteria, Chi-squared test).

### Fasting Plasma Glucose

**Figure 3:**
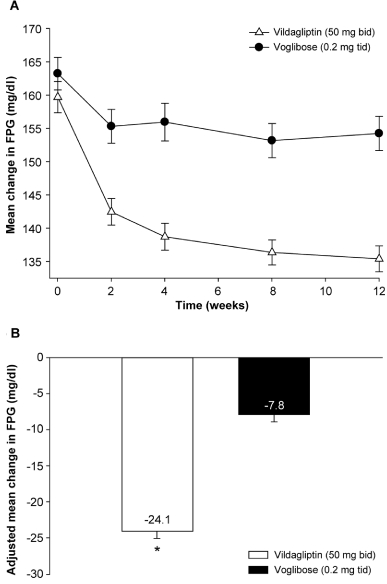
Panel (A): change in mean (±s.e.) fasting plasma glucose (FPG) during the 12-week treatment with vildagliptin 50 mg bid (open triangles) or voglibose 0.2 mg tid (closed circles). Panel (B): least square mean (±s.e.) change in FPG at endpoint under treatment with vildagliptin 50 mg bid (white bar) or voglibose (black bar). 140 and 160 mg/dl are equivalent to 7.8 and 8.9 mmol/l, respectively. *p < 0.001 vs voglibose.

Figure 3 shows reduction in FPG levels. In the vildagliptin group, the onset of reduction was observed as early as week 2 and continued until week 12 (Figure 3A). At endpoint (week 12), the adjusted mean change in FPG reduction from baseline was greater in patients receiving vildagliptin [−1.34 ± 0.09 mmol/l (−24.1 ± 1.54 mg/dl)] compared with those receiving voglibose [−0.43 ± 0.08 mmol/l (−7.8 ± 1.52 mg/dl)] (Figure 3B), with between-treatment difference [0.91 ± 0.12 mmol/l (16.3 ± 2.1 mg/dl)] being statistically significant (p < 0.001).

### Fasting Insulin and C-Peptide

The fasting insulin at baseline was 7.96 ± 0.4 and 8.2 ± 0.4 mU/l in the vildagliptin and voglibose groups, respectively. The absolute change from baseline in fasting insulin was lower in the vildagliptin group (−0.22 mU/l) compared with that of the voglibose group (−0.67 mU/l); and the difference between the groups was not significant (p = 0.154).

The baseline C-peptide levels (2.4 ± 0.1µg/l) and reduction from baseline (∼0.1 µg/l) were similar in the vildagliptin and voglibose groups.

### Meal Test

#### Postprandial Plasma Glucose

**Figure 4:**
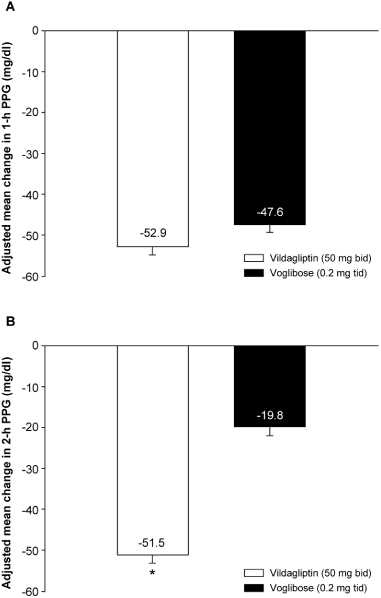
Panel (A): least square mean (±s.e.) change in 1 h postprandial plasma glucose (PPG) at endpoint under treatment with vildagliptin 50 mg bid (white bar) or voglibose (black bar). Panel (B): least square mean (±s.e.) change in 2 h PPG at endpoint under treatment with vildagliptin 50 mg bid (white bar) or voglibose (black bar). Twenty and 40 mg/dl are equivalent to 1.1 and 2.2 mmol/l, respectively. *p < 0.001 vs voglibose.

Figure 4A shows the change in PPG at baseline and at the endpoint. At baseline, the changes in PPG were similar in both groups (Table 1). After 12 weeks of treatment, the change in 1-h [−2.94 ± 0.12 vs. −2.64 ± 0.12 mmol/l (−52.9 ± 2.2 vs. −47.6 ± 2.2 mg/dl)] and 2-h [−2.86 ± 0.13 vs. −1.1 ± 0.13 mmol/l (51.5 ± 2.4 vs. −19.8 ± 2.4 mg/dl)]. PPG from baseline was greater in the vildagliptin group compared with that of the voglibose group. The difference between the groups was significant (p < 0.001) only for the change in 2-h PPG from baseline (Figure 4A, B).

#### Postprandial Insulin

At baseline, 1-h postprandial insulin was 34.5 ± 1.8 and 31.4 ± 1.4 mU/l, respectively, and 2-h postprandial insulin was 31.8 ± 1.7 and 29.7 ± 1.5 mU/l, respectively, for vildagliptin and voglibose treatment groups. The 1-h and 2-h postprandial insulin decreased from baseline in both treatment groups (vildagliptin vs. voglibose: −3.7 ± 0.9 vs. −12.9 ± 0.9;−2.4 ± 1.0 vs. −7.1 ± 1.0; respectively), with difference between them being statistically significant (p < 0.001).

### HOMA-*β* and HOMA-R

Baseline HOMA-*β* and HOMA-R were similar in the treatment groups (vildagliptin, 31.6 ± 1.6 and 3.2 ± 0.2; voglibose, 31.8 ± 1.6 and 3.3 ± 0.2). At endpoint, the adjusted mean change in HOMA-*β* from baseline was greater in vildagliptin (8.2 ± 1.1) compared with that of the voglibose (0.4 ± 1.1) and the difference between them (7.8 ± 1.5) was statistically significant (p < 0.001). HOMA-*β* at endpoint in vildagliptin was 39.7 ± 2.0%. HOMA-R decreased in both groups from baseline at endpoint (vildagliptin, −0.5 ± 0.1; voglibose, −0.40); however, the difference between the groups was not statistically significant (p = 0.367).

### Fasting Lipid and Body Weight

In the FAS population, the baseline triglyceride, total cholesterol, low-density lipoprotein (LDL), high-density lipoprotein (HDL), non-HDL and very low-density lipoprotein (VLDL) in the vildagliptin group were 148.1 ± 6.7, 205.9 ± 2.1, 128.7 ± 2.1, 54.8 ± 1.1, 151.1 ± 2.2, 29.6 ± 1.4 mg/dl and 0.6 mequiv./l, respectively and in the voglibose group the levels were 139.5 ± 7.0, 206.9 ± 2.5, 131.2 ± 2.3, 53.7 ± 1, 153.2 ± 2.5, 27.9 ± 1.4 mg/dl and 0.7 mequiv./l, respectively. The total cholesterol, LDL and non-HDL decreased in the vildagliptin group (−3.1 ± 0.9, −1.9 ± 1.3, −3.2 ± 1.1%), whereas it increased in the voglibose group (4.5 ± 0.9, 9.3 ± 1.3, 6.8 ± 1.1%) and the difference between the treatment groups was significant (p < 0.001 for each). The HDL level decreased with both vildagliptin and voglibose treatment (−1.5 and −0.8%, respectively); however, the difference between them was not statistically significant (p = 0.613).

In the vildagliptin group, the body weight increased by 0.2 kg, whereas in the voglibose group a decrease of 0.8 kg was noted. The difference between the groups was statistically significant (p < 0.001).

### Tolerability

The overall incidence of any AEs, serious adverse events (SAEs) or suspected drug-related AEs was lower in the vildagliptin group compared with that of the voglibose group (Table 3). The incidence of suspected drug-related AEs was lower with vildagliptin compared with that of the voglibose (25.0 vs. 40.6%). The most common suspected drug-related AEs by system organ class was ‘gastrointestinal disorders' (vildagliptin: 11.7%, voglibose: 24.0%). GI AEs were significantly lower in patients treated with vildagliptin (18.6%) compared with that in those treated with voglibose (32.8%) (p = 0.002 Fisher's exact test) (Table 3). Nasopharyngitis was the most common AE in both treatment groups but was not suspected to be as a result of the drugs studied.

**Table 3:** Incidence of AEs, SAEs and hypoglycaemic events in treatment groups (safety population).

Patient	Vildagliptin 50 mg bid (N = 188) n (%)	Voglibose 0.2 mg tid (N = 192) n (%)
All AEs*	115 (61.2)	137 (71.4)
Specific AEs (occurring in >4% of any group)		
Nasopharyngitis	34 (18.1)	32 (16.7)
Constipation	13 (6.9)	13 (6.8)
Flatulence	6 (3.2)	23 (12.0)
Abdominal distension	4 (2.1)	14 (7.3)
Diarrhoea	3 (1.6)	11 (5.7)
Alanine aminotransferase increased	3 (1.6)	11 (5.7)
Suspected drug-related AE†	47 (25.0)	78 (40.6)
Hypoglycaemia	0 (0.0)	1 (0.5)
SAEs	0 (0.0)	4 (2.1)
Discontinuations as a result of AE	4 (2.1)	4 (2.1)
Any GI event (detail below)	35 (18.6)‡	63 (32.8)
Constipation	13 (6.9)	13 (6.8)
Flatulence	6 (3.2)	23 (12.0)
Dyspepsia	5 (2.7)	0 (0.0)
Abdominal distension	4 (2.1)	14 (7.3)
Abdominal pain upper	3 (1.6)	3 (1.6)
Diarrhoea	3 (1.6)	11 (5.7)
Stomach discomfort	2 (1.1)	1 (0.5)
Deaths	0 (0.0)	0 (0.0)

AE, adverse event; GI, gastro intestinal; SAE, serious adverse events.

* A subject with multiple occurrences of an adverse event was counted only once in the adverse event category. When a subject exhibited multiple occurrences of an adverse event in categories of different severity, the subject was counted in the category of worst severity.

† Adverse events occurring in 4% of each group.

‡ p-Value 0.002.

No hypoglycaemic events were observed in the vildagliptin group, whereas one patient in the voglibose group experienced a mild hypoglycaemic event. No incidence of hepatic enzymes abnormalities (≥3 times the upper limit of normal range) for aspartate aminotransferase (AST), alanine aminotransferase (ALT) and alkaline phosphatase (ALP) was noted in any of the groups.

No deaths were reported in this study. There were no SAEs in the vildagliptin group; however, in the voglibose group four patients reported five SAEs (colonic polyp, hand fracture, rib fracture, syncope and asthma) that were not study drug related. Changes from baseline for the haematology, blood biochemistry and urinalysis measurements at endpoint were not clinically significant.

## Discussion

In this 12-week study, vildagliptin 50 mg bid was superior in reducing HbA1c levels compared with voglibose 0.2 mg tid (−0.95 vs. −0.38%; p < 0.001). Greater reduction in the HbA1c levels was evident in all subgroups, including age, gender and BMI, in patients treated with vildagliptin compared with that in the voglibose-treated patient group. Vildagliptin also showed a significant reduction in FPG and 2-h PPG from baseline compared with voglibose (p < 0.001). Vildagliptin was well tolerated with no events of hypoglycaemia and significantly less GI AEs.

The HbA1c reduction observed in this study (0.95%) with vildagliptin 50 mg bid was comparable with the previously reported reduction of 0.92% in Japanese patients with vildagliptin 50 mg bid [16]. As the baseline levels of HbA1c increased from ≤7 to >8, a greater reduction in HbA1c was seen in the vildagliptin 50 mg bid group (-0.56 to −1.35%) compared with that of the voglibose 0.2 mg tid group (−0.27 to −0.49%). Thus in this study, the efficacy of vildagliptin has been showed across a wide range of severity of T2D as defined by HbA1c levels. A higher proportion of patients had HbA1c levels ≤6.5% with vildagliptin compared with that of voglibose (51 vs. 24%; p < 0.001).

The overall efficacy of reduction in HbA1c is essentially the sum of effects on FPG and PPG. In this study, significantly greater FPG reduction from baseline was observed with vildagliptin versus voglibose [1.34 vs. 0.4 mmol/l (24.1 vs. 7.8 mg/dl); p < 0.001]. The reduction in 1-h PPG was similar and that in 2h-PPG levels was significantly greater in the vildagliptin group compared with that in the voglibose group (Figure 4B, p < 0.001). *α*-Glucosidase inhibitors are known to exert glycaemic control by reducing PPG [20,21]; however, in this study vildagliptin showed better PPG reducing ability compared with voglibose. Thus, the overall superior reduction in HbA1c seen in the vildagliptin group compared with that in the voglibose group is essentially because of better reductions in both the FPG and the PPG levels. These findings satisfy the primary goal of diabetes management as set by the IDF guidelines, which is normalization of the glycaemic status with HbA1c, FPG and PPG as markers of disease control [22]. Thus, this study reveals that 24-h plasma glucose control, an important aspect of glycaemic control, would be better in the vildagliptin group compared with that in the voglibose group. A growing body of evidence suggests postmeal hyperglycaemia to be an independent risk factor for the development of macro-vascular disease [23]. Oxidative stress, subclinical inflammation of the vasculature and abnormal carotid intima-medial thickness as an outcome of PPG spikes lead to cardiovascular disease [24]. Thus, treatment to target postmeal hyperglycaemia is important to reduce the risk of both micro- and macro-vascular complications in patients with T2D. Early intensification of treatment could decrease the risk of progression of micro-vascular complications and the damage of *β*-cell caused by chronic hyperglycaemia [22,23]. Controlling postmeal hyperglycaemia with acarbose, an *α*-glucosidase inhibitor, is associated with a significant reduction in the incidence of cardiovascular disease and hypertension [25]. As treatment with vildagliptin 50 mg bid showed greater efficacy in controlling PPG, HbA1c and FPG compared with voglibose, it could be a more appealing alternative for better glycaemic control.

In this study, both treatments resulted in decrease postprandial insulin levels with the extent being lesser in the vildagliptin group compared with that in the voglibose group. Insulin secretion is generally assessed relative to the glucose stimulus. Thus in the absence of any known direct pharmacological effect of voglibose on *β*-cell function, reduced insulin secretion in the face of a small glucose stimulus is not surprising. Vildagliptin, on the other hand, is known to increase *β*-cell function; and this could be the reason for increased insulin secretion observed in the vildagliptin group relative to that seen with voglibose in response to a small glucose stimulus in the present study [26,27]. Furthermore, significant increase in HOMA-*β* in the vildagliptin group compared with that in the voglibose group was observed in the present study, and HOMA-*β* at endpoint in the vildagliptin group reached about 40% which is considered as normal value in Japanese population.

The change in fasting lipid levels from baseline in both treatment groups was not of clinical significance. In previous studies in a wide selection of populations including Chinese, Asian and Non-Indian Asians, treatment with vildagliptin did not lead to any major changes in the fasting lipid profile [28]. In the vildagliptin group, the body weight increased by 0.2 kg, which is not clinically significant, whereas in the voglibose group a decrease of 0.8 kg in body weight was noted, and it was found that there was a statistical significant difference between the groups (p < 0.001). The statistical significance achieved is basically because of the body weight loss in the voglibose group.

Vildagliptin was better tolerated compared with voglibose. No events of hypoglycaemia were reported during the 12 weeks of treatment with vildagliptin, whereas one hypoglycaemic event in one subject was reported in the voglibose group. The incidence of SAEs was 2.1% in the voglibose group, whereas none was reported in the vildagliptin group (0%), and the incidence of AEs (61.2% with vildagliptin vs. 71.4% with voglibose) and suspected drug-related AEs were lower (25.0% with vildagliptin vs. 40.6% with voglibose). There was a lower prevalence of GI AEs on treatment with vildagliptin 50 mg bid (18.6%) compared with that with voglibose 0.2 mg tid (32.8%). The *α*-glucosidase inhibitors are generally associated with GI AEs, which could be a safety concern.

In this study, vildagliptin 50 mg bid was more effective in reducing HbA1c and plasma glucose (FPG and 2-h PPG) with enhanced *β*-cell preservation indicated by HOMA-*β* compared with voglibose 0.2 mg tid. Furthermore, vildagliptin treatment was associated with good tolerability, especially GI tolerability without hypoglycaemia. Thus early treatment with vildagliptin may be a better option for first line of therapy to reduce the hyperglycaemia that characterizes type 2 diabetes; however, the short duration of the study treatment should be taken into consideration while interpreting the results.

## Appendix

The following investigators were involved in the study: T. Miura, Asahikawa-Kosei General Hospital; M. Sekiguchi, Sapporo-Kosei General Hospital; F. Okuguchi, Okuguchi Clinic; K. Yamada, Kenichi Yamada Clinic; T. Nagai, Tomioka General Hospital; N. Yamada, Tsukuba University Hospital; M. Noritake, Noritake Clinic; Y. Suzuki, Asahi General Hospital; T. Imai, Tsuchiura Kyodo General Hospital; K. Ogino, Ogino Hospital; Y. Segawa, Segawa Hospital; S. Mashiba, Mashiba Clinic; M. Kato, Kato Clinic; Y. Aiso, Aiso Clinic; M. Sugawara, Sugawara Medical Clinic; M. Noguchi, Shinagawa East One Medical Clinic; O. Miho, Miho Clinic; T. Kabaya, Minamisenju Hospital; H. Onda, Tokyo Clinical Research Organization for Medicine Clinic; M. Fujita, Shinanozaka Clinic; I. Matsuba, Matsuba Clinic; K. Iemitsu, Kounandai Iemitsu Clinic; M. Tokui, Tokui Clinic; T. Iizuka, Asahi Medical Clinic; T. Takuma, Takuma Saiwai Clinic; M. Nakagawa, Matsumoto Nakagawa Hospital; T. Ohya, Ohya Medical Clinic; G. Watanabe, Watanabe Clinic; N. Takahashi, Takahashi Family Clinic; H. Murase, Daiyukai Clinic; H. Okamoto, Meitetsu Hospital; A. Kashiwagi, Shiga University of Medical Science Hospital; S. Kanada, Heishin-kai OCROM Clinic; S. Kajiyama, Medical Corporation Keisei-kai Kajiyama Clinic; S. Ohashi, Clinic Komatsu; T. Okuno, Nippon Kokan Fukuyama Hospital; Y. Tajiri, Fukuoka City Medical Association Hospital; N. Inokuchi, Inokuchi Internal Medicine Clinic; N. Abe, Abe Diabetes Clinic; M. Hiraga, Nakamura Hospital; S. Nakamura, Heiwadai hospital; T. Kuribayashi, Koga General Hospital; T. Miyakawa, Tama Minami Clinic; O. Tomonaga, Tomonaga Clinic; C. Kondo, Masuko Memorial Hospital; N. Miyamoto, Saino Clinic; M. Kaneshiro, Kaneshiro Diabetes Clinic; T. Miyagishi, Miyagishi Hospital; S. Otabe, Otabe Clinic; K. Shin, Shin Clinic.
